# The quantified immune-aging dysregulation index: a large-language model-powered method for annotating and quantifying systems-level dysregulation

**DOI:** 10.3389/frai.2026.1732901

**Published:** 2026-06-08

**Authors:** George D. Vavougios, Georgios Hadjigeorgiou

**Affiliations:** Medical School, University of Cyprus, Nicosia, Cyprus

**Keywords:** Alzheimer’s disease, artificial intelligence, differential gene expression, gene expression data, inflammation, KNIME analytics platform, large language model, scoring–algorithm

## Abstract

**Background:**

Pathway enrichment analyses are widely used to interpret transcriptomic datasets; however, their outputs typically consist of lists of statistically enriched pathways that require qualitative interpretation and are difficult to compare across biological contexts. Methods of semantic classification that transform enrichment results into quantitative, mechanistically interpretable measures of system-level dysregulation remain underexplored.

**Methods:**

Here, we introduced TENSE (quanTifiEd immuNe-aging dySregulation index), a framework that summarizes pathway enrichment outputs into a quantitative estimate of immune-aging–associated dysregulation. Utilizing a Large Language Model classifier via a KNIME workflow, significantly enriched pathways are semantically classified into five mechanistic categories representing key processes implicated in immune aging, the DIRES scheme: DNA damage (D), DNA repair (R), epigenetic drift (E), inflammaging (I), and nucleic acid sensing (S). These pathway-derived signals are then aggregated into a normalized dysregulation score reflecting the magnitude (TENSE) and distribution (DIRES) of aging-associated processes across biological contexts.

**Results:**

Application of TENSE to transcriptional modules derived from neurodegenerative, radiation-response, and immune activation datasets revealed distinct dysregulation profiles. Alzheimer’s disease–associated modules were primarily characterized by inflammaging signatures, particularly within microglial transcriptional programs, whereas radiation response datasets exhibited dominant DNA damage-related signals. Sepsis-associated gene signatures showed strong inflammatory contributions, producing the highest TENSE values observed. Robustness analysis demonstrated high reproducibility of pathway classification across repeated runs and close agreement between large language model–derived annotations and human consensus scores.

**Conclusion:**

TENSE provides a reproducible and interpretable method for transforming pathway enrichment outputs into quantitative estimates of system-level immune-aging dysregulation. By bridging pathway enrichment analysis and mechanistic interpretation, the framework enables comparative analysis of aging-related biological processes across diverse datasets.

## 1 Introduction

Temporal accounting of biological processes is an essential aspect of system coordination for living organisms, from the cellular level (Gliech and Holland, 2020). Biological time for an organism can be defined as a set of iteratively organized biological processes that maintain cellular and organismal homeostasis throughout its lifespan. Biological time is inescapably linked with physical time (Lestienne, 1988). As organisms progress along physical time, the fidelity of biological processes declines in the human body, defining the process of aging (Tenchov et al., 2024). The loss of biological process fidelity reflects the decay of all cellular systems, from nuclear processes to cellular metabolism and transcellular communication (López-Otín et al., 2023).

One of the more prominent drivers of aging is the progressive accumulation of DNA damage, reflecting a decline in the DNA damage response efficiency (Gorbunova et al., 2007). However, declining repair efficacy leads to the accumulation of additional damage in a feed-forward manner (Schumacher et al., 2021). As a central process in aging, unmitigated DNA damage initiates another feedforward process: inflammation mediated by nucleic acid sensors. This induction, along with potential causes of dysregulated mitigation processes, drives age-related inflammation (Zhao et al., 2023). The loss of genomic stability due to the failure of repair mechanisms thus marks a salient point of no return, where time-dependent disorder prevails over its mitigation (Laffon et al., 2021; Vavougios et al., 2022; López-Gil et al., 2023).

In addition to the idea of inflammaging (Fülöp et al., 2019), these concepts suggest an immune arrow of time—a biological clock that indicates the gradual decline in the accuracy of essential homeostatic processes, observed, as increasing inflammatory tonicity.

The implied parallel between additive disorder in biological systems and aging can also be used to describe the critical role of inflammation in the maturation and development of stem cells (Tatullo, 2024), as well as the critical role of the inflammatory milieu for tissue-specific processes such as synaptogenesis and neurogenesis (Paul et al., 2021). In short, the immune arrow of time may also accelerate purposefully due to physiological processes, as well as secondary to pathophysiological conditions.

An informative approach to identifying systems-level dysregulation is transcriptomic profiling to extract disease-associated gene signatures (Ganesan et al., 2025; Moore et al., 2025; Chen et al., 2026) and the corresponding enriched pathways (Liu et al., 2026; Wang et al., 2026; Song et al., 2026). Gene signature methods quantify the coordinated activity of gene networks, generating weighted scores that can predict outcomes in specific disease contexts (Sparks et al., 2024). Similarly, pathway enrichment analyses identify biological processes that are statistically overrepresented within a dataset, enabling the detection of pathways potentially involved in the underlying biological condition (Vavougios et al., 2022). Similarly, current state-of-the-art applications of LLMs focus on extracting knowledge from gene sets and annotating their function (Wang et al., 2025). While these approaches provide valuable insights into molecular activity and pathway involvement, they primarily address specific research questions within defined biological contexts. Consequently, a methodological gap remains for metrics capable of integrating system-level genomic signals into a quantitative estimate of the magnitude and distribution of dysregulation across predefined biological axes.

In this study, we propose an alternative strategy for annotating pathway enrichment results to extract biologically meaningful information on dysregulation and disease-associated processes. As a proof of concept, we introduce the TENSE (quanTifiEd immuNe-aging dySregulation indEx) scoring framework, designed to quantify immune aging–associated regulatory disorder in biological systems. TENSE functions as an integrative, normalized, systems-level metric that summarizes pathway-derived dysregulation into a single interpretable index, enabling comparative assessment of immune-aging dynamics across datasets and experimental contexts.

TENSE is designed to transform pathway enrichment outputs into a structured representation of immune-aging dynamics by mapping enriched pathways to a set of mechanistically defined biological axes. These axes correspond to key processes implicated in immune aging, namely DNA damage (D), DNA repair (R), epigenetic drift (E), inflammaging (I), and nucleic acid sensing (S), which together form the DIRES scoring scheme. Recent advances in artificial intelligence and computational biology have enabled increasingly sophisticated interpretation of high-dimensional data. These applications include machine learning–based modeling, pathway analysis, and network-level integration approaches. Broadly, these developments illustrate the capacity of AI-driven methods to extract structured insights from complex datasets across diverse scientific domains (Raza and Hanif, 2026; Raza et al., 2026; Hanif et al., 2026; Raza et al., 2025a; Raza et al., 2025b).

Large language models (LLMs) are increasingly integrated into bioinformatics workflows, including assisting with knowledge curation of the Reactome database (Wu et al., 2025). LLMs have been shown to retrieve semantic knowledge to predict regulatory relationships based on provided gene networks, albeit with varying performance (Azam et al., 2024).

In the TENSE workflow, we capitalize LLM capabilities for semantic analysis and classification of enriched pathways into mechanistic aging axes; we then employ them to compute a systems-level dysregulation metric. The LLM core serves as a semantic classifier within this framework, rather than functioning as a biological database. It interprets pathway descriptions and categorizes them into established mechanistic groups: DNA damage, DNA repair, epigenetic drift, inflammaging, and nucleic acid sensing. Pathway nomenclature often contains biological significance that is not consistently captured by simple keyword matching or static database mapping. For instance, pathways such as “HDR through MMEJ” or “OAS antiviral response” necessitate contextual interpretation to appropriately associate them with DNA repair or nucleic acid-sensing processes. The integration of an LLM semantic processing layer, GPT-4.1-NANO, enables scalable and reproducible semantic classification of enriched pathways while retaining mechanistic clarity. It is important to note that the LLM layer GPT-4.1-NANO does not generate biological conclusions; its role is limited to pathway annotation, while the TENSE metric is calculated according to defined quantitative rules. This rigidity also serves to develop TENSE in a relatively controlled data environment while the intended core functionalities are assessed.

TENSE combines the magnitude of enrichment and the pathway contributions along each axis to deliver a normalized quantitative assessment of immune-aging–related dysregulation. With this approach, pathway enrichment outcomes are viewed not simply as lists of significant pathways, but as an integrated metric that represents both the extent and spread of biological disruption among aging-associated processes.

We explore TENSE scoring by reanalyzing relevant published studies and procuring several cases, with a primary focus on Alzheimer’s disease. We demonstrate the utility of the TENSE and its associated measures in annotating pathway enrichment data and the biological context of each studied condition.

## 2 Methods

### 2.1 Theoretical framework

To define TENSE, we consider factors that drive the accumulation of disorder in a biological system as a function of age, herein a cell or tissue, building toward incremental genomic instability (Lemus et al., 2026). For the development of this model, major drivers of aging, adjacent to genomic instability, were considered and modeled as dimensionless variables; Specifically, we considered the equilibrium between DNA damage and repair (Gorbunova et al., 2007; Schumacher et al., 2021; Copp et al., 2023; Vlachogiannis et al., 2023; Whittemore et al., 2019), nucleic acid sensing mechanisms (Paul et al., 2021; Stillman et al., 2024; Dorrity et al., 2024; Hatch and Hetzer, 2015; Miller et al., 2021; Taffoni et al., 2021), inflammaging (Franceschi et al., 2017; Fulop et al., 2023) and epigenetic drift (Bertucci-Richter and Parrott, 2023; Issa, 2014; Bertucci-Richter et al., 2024; Ma et al., 2018).

Although the relationships among these biological processes are complex and involve multiple feedback and regulatory interactions, for the purpose of computing TENSE, their contributions to cumulative dysregulation are modeled as additive. This assumption reflects both the simplifying representation of system-level dysregulation and the statistical framework of pathway enrichment analysis, in which biological pathways are evaluated as independent enrichment events (Nguyen et al., 2019). Consequently, enriched pathways are treated as independent proxies for the underlying biological processes implicated in disease pathogenesis.

Thus, we define the Immune-Entropy Quotient H(t) as a function of aging-related processes:


$$H(t)=\alpha \cdot (\frac{D(t)}{R(t)})+\beta \cdot I(t)+\gamma \cdot E(t)+\delta \cdot S(t)$$

Where:

-
D(t) : DNA damage as a function of time.
-
I(t) : Inflammaging as a function of time.
-
R(t) : DNA repair efficiency as a function of time, with R(t) > 0.
-
E(t) : Epigenetic drift as a function of time.
-
S(t) : Nucleic acid sensing as a function of time.
- Each of *α*,*β*,*γ*,*δ*: weighting parameters reflecting the relative contribution of each term.

The normalized Immune-Entropy quotient would then be calculated with the following formula, when D(t),I(t),R(t),E(t),S(t)is1 :


$$\text{Normalized}\;H(t)=\frac{\alpha \cdot (\frac{D(t)}{R(t)})+\beta \cdot S(t)+\gamma \cdot E(t)+\delta \cdot I(t)}{\alpha +\beta +\gamma +\delta }$$

To calculate normalized H(t) from enrichment datasets, we replace D(t),I(t),R(t),E(t),S(t) proxy scores derived from gene set overrepresentation analyses. To reformulate the function using pathway proxies, we consider the following selection criteria:

1. Proxy pathways for $\frac{D(t)}{R(t)}$ reflect equilibrium between damage and repair, as gene set data typically do not contain DNA damage metadata. Thus, we consider enriched pathways additively rather than as a ratio of damage to response.
2. Proxy pathways for S(t) will include those related to viral infection or intracellular parasites; this choice is intended to capture enriched pathways that may not necessarily reflect infection, but the dysregulation of nucleic acid-sensing/pattern recognition receptors, as reflected by the overrepresented pathways.
3. Proxy pathways for E(t) will include those related to over-represented epigenetic biological functions.
4. Proxy pathways for I(t) will include those related to inflammatory signaling cascades.
5. A notable modeling convention is that, although the conceptual framework regards DNA damage and repair as interconnected processes (D/R), the pathway-based implementation treats them in an additive manner. This approach aligns with the methodology of pathway enrichment analysis, in which enrichment results are generated from distinct pathway annotations, offering independent signals rather than direct assessments of counteracting activities. As a result, pathways associated with damage and repair may demonstrate significant enrichment independently when analyzing a specific gene set. The TENSE (quanTifiEd immuNe-aging dySregulation index) is reformulated as follows:

$$\begin{array}{l} \text{Immune}-\text{Entropy Quotient}={\bar{E}}_{D}\cdot n_{D}+{\bar{E}}_{I}\cdot n_{I}\\ +{\bar{E}}_{R}\cdot n_{R}+{\bar{E}}_{E}\cdot n_{E}+{\bar{E}}_{S}\cdot n_{S} \end{array}$$

Where:

1.
${\bar{E}}_{D},{\bar{E}}_{I},{\bar{E}}_{R},{\bar{E}}_{E},{\bar{E}}_{S}$ represent the arithmetic means of the combined scores obtained via enrichment analysis (Xie et al., 2021) for each proxy pathway for DNA damage, DNA repair, Epigenetic Drift, Inflammaging, and Nucleic Acid Sensing, correspondingly, after weighting for multiple memberships. Specifically, to calculate ${\bar{E}}_{i}$ , i ∈ D, I, R, E, S, while accounting for multiple memberships (i.e., a term belonging to more than one of the DIRES categories), we apply a 1/k weight per combined score for each term, where k = the number of categories each term is a member of. As an example, a pathway identified as belonging to the D, R, S categories (k = 3) would have its combined score divided by 3.
2.
$n_{D},n_{R},n_{E},n_{I},n_{S}$ represents a normalized sum of pathway proxy counts for each of DNA damage, DNA repair, Epigenetic Drift, Inflammaging and Nucleic Acid Sensing, correspondingly. To calculate $n_{i}$ , i ∈ D, I, R, E, S the null hypothesis accepted is that the probability of an enriched pathway is a proxy for at least one of these five categories is 20% (i.e., one in five). The 20% reference probability was used as a neutral null model arising from the five predefined DIRES categories. Under this assumption, in the absence of category-specific enrichment, an enriched pathway assigned to the DIRES framework is expected to have equal prior probability of mapping to any one of the five categories. This assumption is not intended to imply equal biological prevalence of the five processes, but rather to provide a transparent normalization baseline.
3. If the null hypothesis stands, $n_{i}$ is initially calculated as the total number of pathway proxies divided by [0.2 · (Total Number of Enriched Pathways) This formulation produces results that can be interpreted as fold-enrichment, i.e., indicating over- or under-representation based on the ratio observed against expected:

-
$n_{i}=1$ indicates “as expected under the 20% baseline assumption of the model”.
-
$n_{i}>1$ indicates “over-represented”.
-
$n_{i}<1\;\text{indicates}$ “under-represented”.

We again constrain $n_{i}$ by applying a 1/k for each D, I, R, E, S count, to account for multiple memberships in one of the five DIRES categories, leading to standardized values between 0 and 1 for the calculation of TENSE. For example, a pathway scoring in D, R, and S would have a count of 1.

4 If the null hypothesis is rejected, i.e., an enriched pathway does not belong to DIRES, $n_{i}$ = 0, i.e., it does not contribute to TENSE dysregulation.

To provide a standardized estimate of the Entropy Quotient, henceforth the TENSE metric, we divide by ${\bar{E}}_{D}+{\bar{E}}_{I}+{\bar{E}}_{R}+{\bar{E}}_{E}+{\bar{E}}_{S}$ .


$$\begin{array}{l} \text{TENSE}\;\mathrm{as}\;a\;\text{Standardized Immune}-\text{Entropy Quotient}\\ =\frac{{\bar{E}}_{D}\cdot n_{D}+{\bar{E}}_{I}\cdot n_{I}+{\bar{E}}_{R}\cdot n_{R}+{\bar{E}}_{E}\cdot n_{E}+{\bar{E}}_{S}\cdot n_{S}}{\;{\bar{E}}_{D}+{\bar{E}}_{I}+{\bar{E}}_{R}+{\bar{E}}_{E}+{\bar{E}}_{S}} \end{array}$$

6 Standardized TENSE as calculated in step 4 provides values ranging from:

- A minimum of 0, when no enriched pathway belongs to DIRES.
- A maximum of 5, when each of $n_{i}=1$ , after applying the 1/k weight for the five DIRES categories.

To obtain normalized TENSE, producing a range of values between 0 and 1, we divide by the maximum TENSE = 5. Implementation of TENSE and DIRES scoring via an LLM-powered KNIME workflow.

To calculate TENSE in an automated manner, a hybrid semantic – deterministic workflow is designed based on the previously presented theoretical framework and implemented via an LLM-powered KNIME analytical workflow.^1^ The overall architecture uses pathway enrichment analysis via Enrichr to produce combined enrichment scores that adjusts for potential bias in favoring larger length gene sets in gene set libraries (Chen et al., 2013). Thus, Enrichr combined scores provide weighted contributions to pathways by incorporating both statistical significance and enrichment magnitude. Notably, while Enrichr combined scores provide a useful measure of pathway relevance within a given analysis, they are not inherently comparable across datasets. The TENSE framework addresses this limitation by normalizing pathway contributions, enabling comparison of the relative structure of dysregulation across biological contexts. Within the TENSE framework, these scores are utilized to maintain the relative importance of pathways within enrichment analyses, followed by normalization across DIRES categories. Consequently, TENSE characterizes the distribution of dysregulation in each dataset rather than depending on direct comparison of raw enrichment scores from independent analyses.

Pathways included in each DIRES category were selected for their established mechanistic links to biological processes relevant to immune aging, such as genomic instability, DNA repair capacity, epigenetic regulation, chronic inflammatory activation, and nucleic acid sensing. Enrichment analyses utilized Enrichr,^2^ which offers curated pathway annotations derived from recognized biological databases including Reactome, KEGG, and Gene Ontology. These curated pathways enable enriched terms to be mapped to mechanistically interpretable biological processes, a requirement of the DIRES framework underlying the TENSE metric. Conversely, clustering approaches like MCODE (Zhou et al., 2019) in Metascape identify densely connected gene clusters based on network topology. However, while useful for exploratory network analysis, these clusters are dependent on individual datasets and may not align with predefined biological mechanisms.

**Figure 1:**
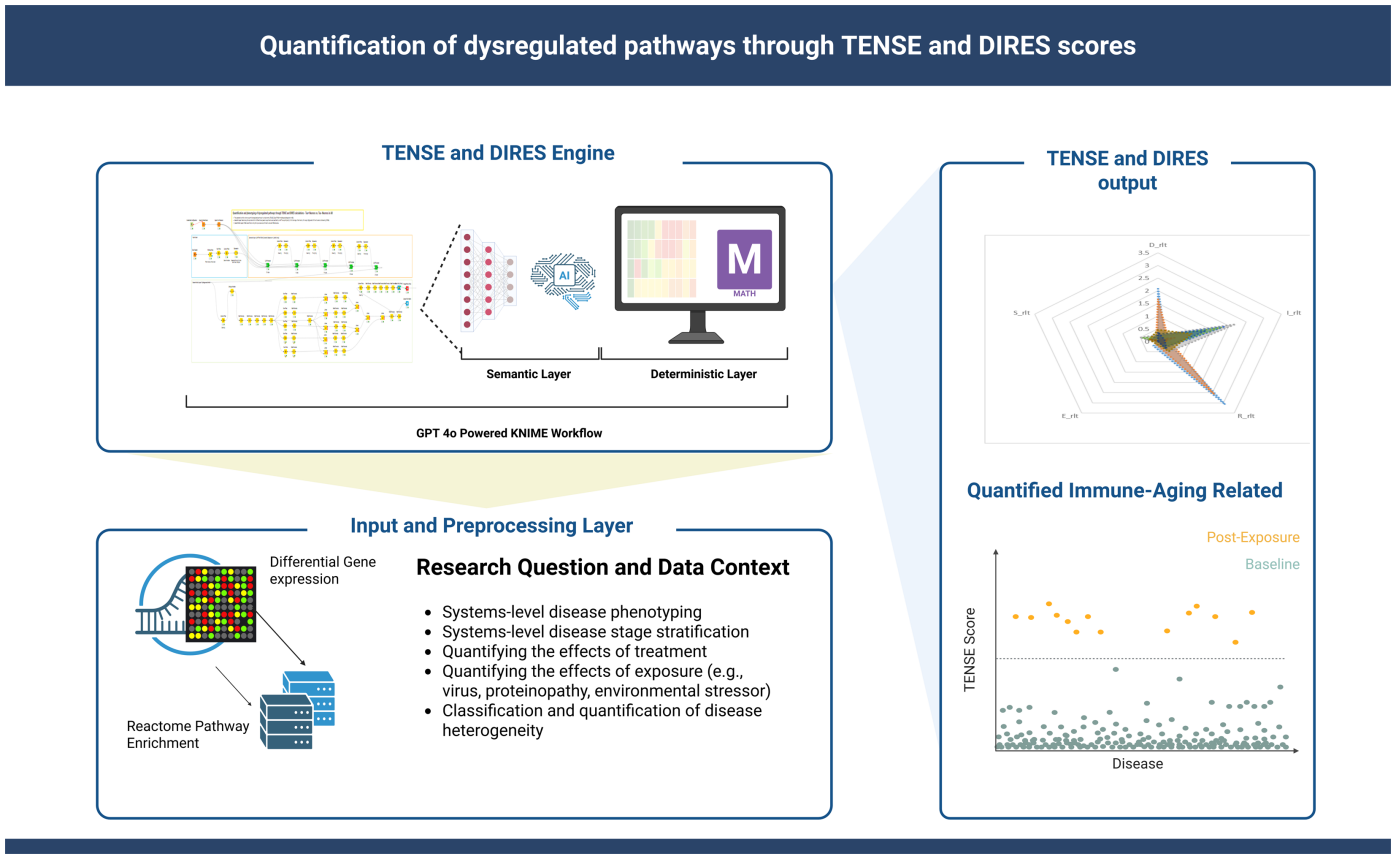
An overview of the study design, including the calculations for TENSE (quantified immune-aging dysregulation index) and DIRES (DNA damage, inflammaging, DNA repair, epigenetic drift, sensing mechanism). The workflow accepts input in the form of significantly enriched Reactome pathways from RNA sequencing experiments designed to address specific research questions. The TENSE and DIRES engine is a hybrid semantic – deterministic workflow in which natural language processing is used to score Reactome pathways according to the DIRES scheme (semantic layer) and then calculate TENSE (deterministic layer). Created in BioRender (https://biorender.com) by GV and licensed under CC BY 4.0.

Because the TENSE framework requires consistent mechanistic axes across datasets to quantify immune-aging–associated dysregulation, curated pathway annotations were preferred over topology-based clustering methods. Subsequently, enriched terms and combined scores are then analyzed by an LLM-powered KNIME workflow to provide DIRES and TENSE scoring. KNIME is an open-source workflow management platform with a graphical user interface that enables users to design and implement sophisticated data science pipelines (Song et al., 2026). In recent updates, KNIME has incorporated AI nodes to facilitate the integration of large language models, further empowering designed workflows. Figure 1 presents an overview of the study design.

### 2.2 Semantic layer: LLM-powered scoring of enriched biological processes

For the semantic layer of the workflow, natural language processing of enriched processes was performed via an API node using GPT-4.1-nano. GPT-4 has been previously utilized for processing the Reactome DB (Tiwari et al., 2023) and biomedical literature (Poretsky et al., 2025), and thus the GPT-4.1-nano^3^ was selected. GPT-4.1-nano is one of the default choices provided by the OpenAI LLM node in KNIME. The main advantages it provides are cost-effectiveness and speed according to its specifications.^4^ The use of GPT-4.1-nano is intended as a proof of concept and relies on at least one use case involving GPT-4 processing of Reactome data. The application of other LLMs, newer versions of GPT-4.1-nano (e.g., GPT-5-nano), as well as manual pathway annotation and TENSE calculation, is equally feasible.

In KNIME, GPT-4.1-nano is accessed via a unique API key, which is applied to an authenticator node and connected to an OpenAI LLM selector node. For this proof-of-concept application, GPT-4.1-nano was used with default settings, with a sampling temperature of 0.2 and a maximum response length of 200 tokens. Notably, sampling temperatures typically range from 0.0 to 1 (and above), with higher values allowing for creative responses, whereas lower temperatures, e.g., 0.0 to 0.3, correspond to answers with greater precision and are more appropriate when exact answers are required (Hong et al., 2025).

To score enriched pathways by DIRES, LLM prompter nodes were connected serially to GPT-4.1-nano and subsequently prompted via Expression Nodes to score significantly enriched terms (provided by input nodes; defined by a false discovery rate <0.05). The prompts are available in the online version of the workflow and from Supplementary File 1. An example of a D node prompt is the following, delivered to an LLM prompter node via a KNIME Expression Node:

“You are a strict binary classifier.

OUTPUT CONTRACT (MANDATORY):

- Output exactly N lines, where N = number of input terms.

- Each line MUST be exactly ONE character: either 0 or 1.

- No spaces, no punctuation, no text.

- Multi-digit outputs like 10, 01, 00 are INVALID.

If any rule is violated, correct it before responding.

SELF-CHECK (DO NOT PRINT):

Verify: (1) line count == N, (2) each line is exactly one character and is 0 or 1.

TASK:

Return 1 ONLY if the pathway term directly reflects DNA damage, genotoxic stress, or genomic instability (damage presence/consequences). Else 0.

DEFINITION (DNA damage):

Processes indicating DNA lesions, genome instability, or cellular responses that signal damage presence (NOT the repair execution mechanisms).

POSITIVE ANCHORS (strong evidence to score as 1):

- DNA damage

- genomic instability

- genotoxic stress

- DNA lesion(s)

- replication stress (as damage/stress)

- DNA strand break(s) (if not explicitly ‘repair’)

- p53-mediated damage response / p53 signaling in response to damage

- ATM/ATR checkpoint signaling in response to DNA damage (signaling/checkpoint, not repair execution)

- oxidative DNA damage / ROS-induced DNA damage

OVERRIDE RULE (ALWAYS 1 if present — highest priority):

If the term contains any of these tokens (case-insensitive), output 1:

DNA damage, genotoxic, genomic instability, replication stress, DNA lesion, strand break, double-strand break (DSB) (unless explicitly ‘repair’), single-strand break (SSB) (unless explicitly ‘repair’), p53 (when clearly damage-related), ATM, ATR (when clearly damage/checkpoint related).

NEGATIVE ANCHORS (strong evidence to score as 0):

- DNA repair mechanisms

- RNA damage response mechanisms

- Nucleic acid sensing mechanisms

- Immune processes not directly triggered by DNA damage

- General cell cycle/replication without damage context

If uncertain, output 0.

INPUT TERMS (one term per line):”

The output provided at the end of this process is a table containing the DIRES scores before weighting for multi-category membership (Table 1).

**Table 1:** An example of DIRES output following serial processing and scoring.

Term	D_count	Ι_count	R_count	E_count	S_count
Immune System	0	0	0	0	0
Innate Immune System	0	1	0	0	0
Neutrophil Degranulation	0	1	0	0	0
Keratinization	0	0	0	0	0
Cytokine Signaling in the Immune System	0	1	0	0	0
Signaling by Interleukins	0	1	0	0	0
RAC1 GTPase Cycle	0	0	0	0	0

The terms are Reactome pathways, whereas the count columns score binary for DNA damage (D_count), Inflammaging (I_count), DNA Repair (R_count), Epigenetic drift (E_count), and Nucleic Acid Sensing (S_count).

This scoring scheme is performed independently for each D, I, R, E, S category and serially, D ➔ I ➔R ➔E ➔S, using a dedicated LLM prompter node and prompt. This design choice was intended to provide greater precision for each category while providing a dissectible architecture for troubleshooting and further development. The final DIRES scoring table is then submitted to the deterministic layer for further calculations.

### 2.3 Deterministic layer: calculating TENSE via DIRES

The deterministic layer utilizes multiple KNIME nodes to calculate TENSE. To improve interpretation, we also provide normalized values for TENSE and for each DIRES category. This design choice enables easier interpretation as TENSE can score from 0 to 1 following normalization (i.e., 0–100% dysregulation), rather than 0–5 as a standardized value. To normalize TENSE’s values, we divide the derivative score from steps 1–5 by 5, i.e., the maximum standardized value of TENSE.

Specifically, the following step-wise procedure is followed to calculate TENSE from the DIRES pivot table.

1. Accepts input from the Semantic Layer in the form of the DIRES pivot table, containing the scores of each term in the D, I, R, E, S categories.
2. Calculates the sum of each D, I, R, E, S via Math Formula KNIME nodes.
3. Calculates the arithmetic mean of the combined scores (provided by Enrichr) for each term scoring in D, I, R, E, S for each category via Math Formula KNIME nodes.
4. Calculates the standardized D, I, R, E, S scores by dividing the sum of proxy pathways in each category by [0.2 · (Total Number of Enriched Pathways)].
5. Calculate TENSE as a standardized score by applying 1/k weighting as previously described.

As an overview of the workflow, the following process is followed to calculate DIRES and TENSE:

1. LLM-based scoring of enriched pathways for membership in D, I, R, E, S.
2. Calculation of the standardized D, I, R, E, S scores by dividing the number of proxy pathways in each category by [0.2 · (Total Number of Enriched Pathways)].
3. Calculation of the arithmetic means of the combined scores for D, I, R, E, and S proxies.
4. Calculate the standardized TENSE by $\frac{{\bar{E}}_{D}\cdot n_{D}+{\bar{E}}_{R}\cdot n_{R}+{\bar{E}}_{E}\cdot n_{E}+{\bar{E}}_{I}\cdot n_{I}+{\bar{E}}_{S}\cdot n_{S}}{\;{\bar{E}}_{D}+{\bar{E}}_{R}+{\bar{E}}_{E}+{\bar{E}}_{I}+{\bar{E}}_{S}}$ .
5. Normalize TENSE score to a range of values between 0,1.

When none of the DIRES categories have enriched pathways, all components of the TENSE score become zero, resulting in a zero denominator. In the KNIME workflow, this leads to a missing value. To ensure clarity and consistency, these cases are deliberately assigned a TENSE value of 0. This approach indicates that there is no DIRES-associated dysregulation in the analyzed pathways, instead of representing a numerical calculation from non-zero values. Therefore, a TENSE score of 0 should be understood as an absence of immune-aging–related dysregulation within the boundaries of this framework.

### 2.4 Quality control and accuracy

#### 2.4.1 Eidetic layer: restricting the black box effect of the LLM-scoring process

For quality control and to reduce black-box effects, we established a parallel explanatory layer that provides insight into the LLM scoring process and decision-making. This explanatory layer uses the same prompts, albeit instructed to provide a rationale rather than a binary classifier exclusively. This layer can be connected to the main classifier to provide insight into the DIRES scoring process.

As an example, for the first term in Table 1, the Eidetic Layer provides the following exchange when checking the term “Immune System” for membership in D:

#### 2.4.2 GPT-4.1-nano response

- *The term “Immune System” is primarily associated with the body’s defense mechanisms against pathogens, including bacteria, viruses, and other foreign substances. While the immune system can sometimes be involved in processes related to DNA damage—such as immune responses to DNA damage or immune cells in recognizing damaged cells—it is not inherently or exclusively a DNA damage process.*
- Therefore, since “Immune System” broadly pertains to immune responses rather than directly to DNA damage processes, the appropriate response is: **0**.
**Rationale:** The immune system’s primary functions are immune defense, immune regulation, and pathogen recognition, not DNA repair or damage processes.

We subsequently assess the rationale for each answer to ensure consensus between manual scoring by the author and GPT-4.1-nano. Notably, this example, “Immune System,” is a broad term that does not specify processes that map to inflammaging and, by default, is not intended to score in the I category to avoid artificial inflation.

### 2.5 Accuracy and precision metrics with reference to a human consensus

#### 2.5.1 Theoretical approach

To address LLM non-determinism, i.e., the variability between repeated responses for the same prompt (Shusterman et al., 2025), we performed multiple (*n* = 100) sequential runs of a single dataset with at least one valid term for each category in DIRES.

We then extract TENSE values for each run and calculate mean TENSE±SD and 95% Confidence interval (CI). Subsequently, we score the dataset using DIRES as a human-consensus and provide a human-consensus TENSE value.

Subsequently, we use the latter value to calculate the following metrics:

- Bias of the Mean (BM): Mean TENSE (LLM) − Human Consensus TENSE (Wilmoth, 2012; Carstensen, 2010).

$$\text{Relative Bias of the Mean (RBM):}\frac{\mathrm{BM}}{\text{Human Consensus TENSE}}\;$$

$$\text{Coefficient of variation (CV):}\frac{\mathrm{SD}}{\text{Mean TENSE}\;(\mathrm{LLM})}$$

#### 2.5.2 Implementation dataset

To assess TENSE’s accuracy and precision, we utilize a transcriptomic module from Sparks et al. (2024) study. In brief, Sparks et al. (2024) performed weighted gene co-expression network analyses (WGCNA) of whole-blood transcriptomics from 228 patients of 22 monogenic immune diseases and 42 age- and sex-matched healthy participants. WGCNA identified 12 transcriptional modules that are differentially enriched for immune processes and cells. Among available TMs, we select TM1, which is enriched for Type I interferon responses. The rationale for this selection is that Type I interferon signaling and related pathways are expected to map broadly to DIRES categories (Cao, 2022; Papatriantafyllou, 2013). This will ensure that a non-zero TENSE score can be calculated and used to identify oscillations caused by LLM stochasticity.

**Figure 2:**
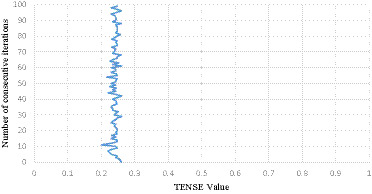
TENSE value calculations over 100 iterations of the KNIME workflow on the Interferon Signaling transcriptional module.

TENSE is calculated as described above via a looped version of the TENSE workflow, utilizing the Counting Loop Start and Loop End nodes for *n* = 100 iterations of the workflow; These workflow iterations furthermore correspond to 5 prompt iterations for each of DIRES categories per significantly enriched pathway (n_pathway_ = 38), for a total of 100x5x38 = 1,540 total prompt iterations utilizing GPT-4.1-nano via LLM prompter nodes. (Supplementary File 1). TENSE scores produced via this process (Mean TENSE LLM = 0.242 ± 0.015, 95% CI:0.240–0.244) demonstrated high concordance with human consensus (BM = −0.006; RBM = −2.7%) and stable performance across repeated runs (SD = 0.015; CV = 4.24%; Table 2). The TENSE values produced by the workflow across 100 runs are available as Supplementary material 1 (Figure 2).

**Table 2:** TENSE calculations following 100 iterations of GPT-4.1-nano.

Metric	Value
Reference TENSE	0.248
LLM mean TENSE	0.242
Standard deviation	0.015
95% confidence interval (Half-Width)	0.002
Bias of the mean (BM)	−0.006
Relative BM	−0.027
Coefficient of variation (CV)	0.042

Reference TENSE is calculated by a human consensus, i.e., by annotating each pathway as DNA damage, Inflammaging, DNA repair, epigenetic drift, and then manually calculating TENSE. LLM: large language model, here GPT-4.1-nano.

Subsequently, we leveraged Reactome Pathways from TM10, a transcriptional module enriched for housekeeping cell signaling pathways, which, by design, are not expected to score in DIRES. The workflow loop produced a consistent TENSE value of 0 across 100 independent workflow iterations, indicating no spurious DIRES activation and demonstrating high classifier specificity.

#### 2.5.3 Interoperability and modularity

To ensure TENSE’s application beyond the manual version of the workflow, we implemented Anthropic’s Claude Haiku 4.5 (Available as CLAUDE-HAIKU-4-5-20251001 from the KNIME LLM nodes) as an alternative LLM classifier for the semantic layer at the same sampling temperature T = 0.2, as previously described. The Haiku model was selected as relatively equivalent to GPT-4.1-nano in terms of cost efficiency for API calls, accuracy, and speed (Available from: https://platform.claude.com/docs/en/about-claude/models/overview; Accessed on March 9th, 2026).

TM9, enriched for RNA and RNA-related processes, was utilized as the comparison dataset. Genes comprising the module underwent the process described previously to produce a Reactome term list and associated Enricher-derived metrics.

**Figure 3:**
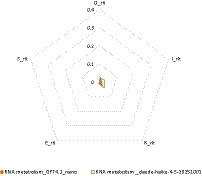
The DIRES radar plot on the RNA metabolism transcriptomic modules by Chen and colleagues; note that modules Yellow and Pink produce a zero TENSE score. The “_rlt” suffix denotes the utilization of relative scores (i.e., $n_{i}$ , i ∈ D, I, R, E, S) for each DIRES category.

Using fixed prompts for both LLM classifiers, Claude Haiku produced comparable results (Table 3 and Figure 3), indicating that the TENSE / DIRES workflow is interoperable between LLMs.

**Table 3:** Head-to-head comparison between GPT4.1-nano and Claude Haiku 4.5.

Reactome term	mODEL	D_rlt	I_rlt	R_rlt	E_rlt	S_rlt	TENSE
RNA metabolism	GPT-4.1-nano	0.044	0.013	0.033	0.013	0.002	0.025
RNA metabolism	claude-haiku-4-5-20251001	0.022	0.030	0.041	0.009	0.001	0.041

The “_rlt” suffix denotes the utilization of normalized relative scores (i.e., $n_{i}$ , i ∈ D, I, R, E, S normalized bounded by 0,1) for each DIRES category.

Claude Haiku produced several formatting errors that skewed DIRES; specifically, instead of binary classification, response cells contained text with descriptive answers. An example follows:

- Input: Pathway to be assessed: PIP3 Activates AKT Signaling:
- Response: *Here are the results for the terms in “PIP3 Activates AKT Signaling”: PIP3: 0, Activates: 0, AKT: 0, Signaling: 0*

For the calculation of TENSE using Claude Haiku 4.5, formatting errors were treated as missing values; GPT-4.1-nano did not produce format errors. Despite fail-safes and consistent performance by GPT-4.1-nano, these format errors in Claude Haiku 4.5 response structure may plausibly reflect the result of prompt structure and its bias toward GPT-4.1-nano nativity. Further refinement with Claude was not pursued beyond the feasibility phase, in favor of GPT-4.1-nano’s performance on the given task and, by extension, API cost-efficiency.

To assess the robustness of the workflow in scoring a predetermined biological concept, we implemented an alternative/perturbed DIRES scheme where:

- DNA damage and DNA repair mechanisms are aggregated in the D node.
- Cellular signaling replaces DNA Repair in the R node.
- Cellular metabolism replaces Epigenetic in the E node.

We then reanalyzed TM10, enriched for cellular signaling, and with a zero TENSE score. The perturbed TENSE score was 0.246 (24.6%), with cellular signaling cascades inflating the novel R node. We conclude that the workflow is adequately modular in the sense that it can serve biological concepts beyond those intended by TENSE’s design, e.g., function as a cell signaling/metabolic/stress classifier based on the design of the semantic layer.

### 2.6 Access and availability

The current version of the TENSE classifier workflow is available via the KNIME community hub under/gvavou. An individual API key, or manual DIRES annotation of the datasets to be analyzed, is required to run each iteration of the workflow.

## 3 Results

### 3.1 Use cases for TENSE and DIRES annotation in the literature

Following the construction of the TENSE engine, we examine the utility of both TENSE and DIRES scores by annotating published datasets in various settings as use cases. We examine the TENSE/DIRES annotations in prior studies to determine whether they adequately capture biological information and offer further insight, as well as a novel means of presenting that information. Raw data usage from referenced studies, Enrichr access to Reactome,^5^ KNIME workflow utilization,^6^ and API Calls to GPT-4.5-nano (via KNIME AI integration) were performed on March 9th, 2026.

#### 3.1.1 Use case 1: annotating Alzheimer’s disease immune aspects on neuron and microglial gene signatures

##### 3.1.1.1 Background and rationale

For the first use case, we will annotate cell-specific gene signatures associated with Alzheimer’s disease diagnosis and pathology. We will furthermore examine the concordance between Neuron- and microglia-specific gene signatures across two distinct studies (Chen et al., 2022; Morabito et al., 2020). Specifically, we utilized a Neuronal-derived consensus gene module from medial temporal gyri from Alzheimer’s disease compared to control, as reported by Chen et al. (2022). Briefly, Chen and colleagues performed weighted gene co-expression network analysis (WGCNA) on 10,000 highly variable genes, identifying eight co-expression gene modules, four of which (yellow, brown, pink, and turquoise) showed to correlate with AD pathology.

DIRES and TENSE annotations on Chen et al.’s dataset were compared to annotations on corresponding neuron-enriched (Consensus Module 1; CM1) and microglia-enriched (Consensus Module 8; CM8) AD-associated gene modules reported by Morabito et al. (2020). In their study, Morabito et al. (2020) reported on WCGNA-derived consensus gene modules that were correspondingly microglia- and neuron-specific, and associated with Alzheimer’s disease.

Finally, we aimed to determine whether TENSE and DIRES annotations would be conceptually concordant with data reported by Das et al. (2024) by comparing the response transcriptomes between Αβ plaques vs. neurofibrillary tangles, which revealed inflammation as the most salient component.

##### 3.1.1.2 Approach and results

**Figure 4:**
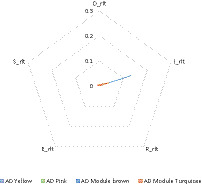
The DIRES radar plot on the four AD-related transcriptomic modules by Chen and colleagues; note that modules Yellow and Pink produce a zero TENSE score. The “_rlt” suffix denotes the utilization of normalized relative scores (i.e., $n_{i}$ , i ∈ D, I, R, E, S normalized bounded by 0,1) for each DIRES category.

**Figure 5:**
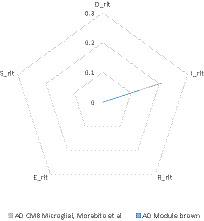
The DIRES radar plot on the AD-related brown module by Chen and colleagues, and the corresponding AD microglial module by Morabito and colleagues. The “_rlt” suffix denotes the utilization of normalized relative scores (i.e., $n_{i}$ , i ∈ D, I, R, E, S normalized bounded by 0,1) for each DIRES category.

**Figure 6:**
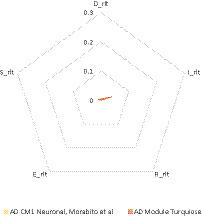
The DIRES radar plot on the AD microglial gene module by Chen and colleagues, and the corresponding AD microglial module by Morabito and colleagues. The “_rlt” suffix denotes the utilization of normalized relative scores (i.e., $n_{i}$ , i ∈ D, I, R, E, S normalized bounded by 0,1) for each DIRES category.

**Figure 7:**
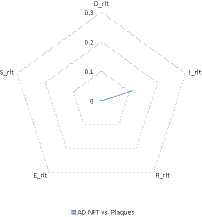
The DIRES radar plot for the “Aβ plaques vs. neurofibrillary tangles” gene signature extracted from Das and colleagues, indicating a predominantly inflammatory component. The “_rlt” suffix denotes the utilization of normalized relative scores (i.e., $n_{i}$ , i ∈ D, I, R, E, S normalized bounded by 0,1) for each DIRES category.

We performed enrichment analysis for each pair of gene modules using Enrichr and identified significantly over-represented pathways from Reactome. We replicated the steps described previously. Subsequently, we obtain the following values for TENSE:

- For the AD-related modules by Chen and colleagues, we obtain the following values: TENSE_yellow_ = 0, TENSE_pink_ = 0, TENSE_brown_ = 0.131 (13.1%), and TENSE_turquoise_ = 0.012 (1.2%).
- For the neuronal module (CM1), we obtain TENSE_CM1_ = 0.022 (2.2%) and for the microglial module (CM8) TENSE_CM8_ = 0.208 (20.8%) (Figures 4–7).

#### 3.1.2 Use case 2: annotating the differential transcriptomic response of radiation-resistant and radiation-sensitive cells exposed to 4 Gy radiation at 4- and 12-h post-exposure

##### 3.1.2.1 Background and rationale

Radiation induces both genotoxic stress and inflammation in affected cells; contrary to annotated data from AD, data from cellular irradiation inform us on the temporally resolved effects of a well-defined exposure on the transcriptome. To approach this research question, we utilized data available from Borràs-Fresneda et al. (2016). Their study reported differences in gene expression between radiosensitive (RS) and radioresistant (RR) cell lines at various time points after irradiation at a dose of 4 Gy.

##### 3.1.2.2 Approach and results

To obtain DIRES and TENSE annotations, we replicate the steps described previously. Subsequently, we obtain the following values for TENSE:

- For the radiosensitive line, TENSE = 0.044 (4.4%) at 4 h vs. TENSE = 0.364 (36.4%) at 12 h.
- For the radioresistant line, TENSE = TENSE = 0.2 (20%) at both 4 h and 12 h.
- We then calculate the differences between baseline and post-exposure TENSE scores.
- We define the difference as ΔTENSE = TENSE_post-exposure_ - TENSE_baseline_ for both scenarios.
- In the Radiosensitive line, ΔTENSE = 32%, whereas in the radioresistant line, ΔTENSE = 0.

**Figure 8:**
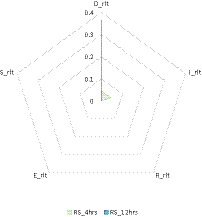
The DIRES radar plot demonstrates a transient inflammatory component evident at 4 h and absent at 12 h, while the DNA damage component remains active. The “_rlt” suffix denotes the utilization of normalized relative scores (i.e., $n_{i}$ , i ∈ D, I, R, E, S) for each DIRES category. Gy: Gray, RS: Radiosensitive; RR: Radioresistant.

**Figure 9:**
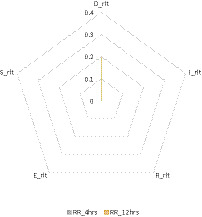
The DIRES radar plot demonstrates an invariant polarization toward DNA damage pathways at 4 h and 12 h. The “_rlt” suffix denotes the utilization of relative scores (i.e., $n_{i}$ , i ∈ D, I, R, E, S) for each DIRES category. Gy: Gray, RR: Radioresistant.

Figures 8, 9 display Radar plots for DIRES scores for the radiosensitive and radioresistant lines, respectively. DIRES annotation suggests that the radiosensitive line exhibits an inflammatory component at 4 h, which is absent at 12 h post-exposure. Conversely, the radioresistant line’s transcriptome indicates invariant activation of repair mechanisms at both time points, and the absence of a detectable inflammatory component.

DIRES annotation outlines a sustained inflammatory and DNA damage component for radiosensitive cells, whereas radioresistant cells display an invariant DNA repair component. Differences in TENSE scores indicated a more dynamic utilization of cellular processes in radiosensitive cells and a robust yet invariant utilization for radioresistant cells, polarized toward repair.

These findings are in agreement with the findings of the original study, where differences in DNA repair capacity and mitigation of radiation-induced inflammation Yahyapour et al. (2018) between the two cell types was outlined. With our approach, both TENSE and DIRES serve to quantify these changes beyond the study of differentially expressed genes or pathways of interest. The concept of ΔTENSE, i.e., the difference between TENSE values at baseline and post-exposure, is useful for distinguishing between stable and dynamic processes.

#### 3.1.3 Use case 3: TENSE and DIRES as descriptors of immune gene network signatures

##### 3.1.3.1 Background and rationale

Conserved gene networks have previously been linked to impaired regulation of the host response to infection, and their activity is often captured by distinct gene signatures that link gene activity with clinical outcomes. One such signature, the 42-gene Severe-or-Mild (SoM) signature, was found to be associated with several risk factors for poor outcomes from infection, mortality, and capture the response to treatment Ganesan et al. (2025). Similar approaches posit that deviations from health, including aging and disease, have a common systemic immune dysregulation footprint. The 150-gene signature Immune Health Metric (IHM) score represents one such effort to capture this shared influence (Sparks et al., 2024). Specifically, the IHM correlates with aging in healthy individuals and provides differential values between healthy individuals and those with monogenic diseases. In this use case, we explore TENSE and DIRES as descriptors of SoM and IHM scores. To calculate TENSE and DIRES, we assume that the SoM and IHM genes are differentially expressed, map enriched processes to DIRES, and calculate TENSE for each SoM and IHM score.

##### 3.1.3.2 Approach and results

As described in use case 1, replicating the procedures established to calculate DIRES and TENSE, we determine the following TENSE values. Notably, as IHM is calculated as the difference between the geometric means of two gene sets (Sparks et al., 2024), (herein referred to as IHM-A and IHM-B), we calculate TENSE and DIRES for each.

**Figure 10:**
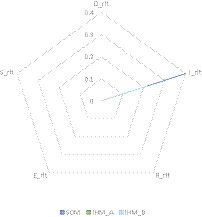
The DIRES radar plot demonstrates an invariant polarization toward inflammaging for the IHM-B and SoM signatures, whereas IHM-A signatures do not score in any DIRES categories, resulting in a TENSE = 0. The “rlt” suffix denotes the utilization of relative scores (i.e., $n_{i}$ , i ∈ D, I, R, E, S) for each DIRES category. IHM: Immune ealth etric; SOM: evere-or-mild index.

- For SoM: TENSE_SoM_ = 0.4 (40%).
- For IHM-A: TENSE_IHM-A_ = 0.
- For IHM-B: TENSE_IHM-B_ = 0.217 (21.7%).
- For IHM, we furthermore calculate the ΔTENSE = TENSE_IHM-A_ – TENSE_IHM-B_ = −21.7% (Figure 10).

## 4 Discussion

This study introduces TENSE (quanTifiEd immuNe-aging dySregulation indEx), a framework that turns pathway enrichment results into a quantitative, system-level measure of immune-aging–related dysregulation. Instead of generating typical lists of significant pathways for qualitative interpretation, TENSE integrates signals from multiple pathways into key biological categories, summarizing them as an understandable dysregulation profile. As a result, it offers a higher-level overview of enrichment results, making it easier to compare biological situations based on the degree and type of aging-related regulatory imbalance present. The classification used in TENSE proved reasonably reliable, giving consistent results across repeated tests and closely matching human expert assessments. Similar outcomes were observed with alternative large language models such as Claude Haiku, suggesting that the underlying classification task is well-structured and produces stable results across different tools. Each of the five DIRES axes was found in at least one of the analyzed datasets, indicating that TENSE captures a variety of dysregulation patterns rather than being limited to just one category. Overall, these findings support TENSE as a reproducible and easy-to-interpret approach for converting complex enrichment outputs into meaningful estimates of immune-aging dysregulation.

### 4.1 Critical analysis of TENSE use cases

Application of the TENSE framework across transcriptional modules and experimental contrasts revealed distinct patterns of aging-associated immune dysregulation enrichment patterns across biological contexts. Within Alzheimer’s disease (AD) modules derived from WGCNA analysis, TENSE values were primarily driven by the inflammaging axis, with the strongest contributions observed in the CM8 microglial module (TENSE = 0.208/20.8%) and the brown module (TENSE = 0.131/13.1%). This pattern is consistent with extensive evidence implicating microglial activation and chronic innate immune signaling as key contributors to AD pathology. Conversely, neuronal modules such as CM1 exhibited relatively low TENSE values, with minor contributions from both inflammaging and nucleic acid sensing pathways, suggesting that immune-aging–related dysregulation is less pronounced within neuronal transcriptional programs. Similarly, the comparison between neurofibrillary tangles and plaques demonstrated moderate TENSE values largely attributable to inflammaging, a finding corroborated by the role of inflammatory signaling in AD-associated transcriptional dysregulation.

Outside the AD context, distinct enrichment patterns emerged. In the radiation response experiments, TENSE values were dominated by the DNA damage axis, particularly in the radiosensitive condition at 12 h (RS_12h; TENSE = 0.364/36.4%). This observation is consistent with the induction of genomic instability and tandem DNA damage–response pathways following irradiation, representing expected transcriptional responses to ionizing radiation (Miyake et al., 2019). Conversely, the sepsis-associated SOM gene signature yielded the highest TENSE value observed in the dataset (0.4/40%), driven entirely by the inflammaging axis, highlighting the extensive inflammatory activation characteristic of systemic infection. The Immune Health Metric gene signatures further illustrated this contrast: while IHM_A showed no detectable DIRES contributions, IHM_B exhibited a strong inflammaging signal (TENSE = 0.217/21.7%), suggesting that subsets of immune health–associated gene programs may capture inflammatory activation states. Notably, ΔTENSE for the two IHM signatures is negative, a finding that is in accordance with SoM and IHM scores, as expected to be inversely correlated (Ganesan et al., 2025): DIRES annotation suggests that high IHM reflects a shift from IHM-B contributors, i.e., Inflammaging and Nucleic Acid Sensing (higher TENSE values) to IHM-A contributors, primary nucleic acid sensing mechanisms, and those associated epigenetic drift at a quiescent state (lower TENSE values).

Finally, the TM10 RNA metabolism module exhibited low but distributed contributions across multiple DIRES axes, including DNA damage, DNA repair, epigenetic drift, and inflammaging, producing a comparatively modest TENSE value. This pattern suggests diffuse engagement of multiple regulatory processes rather than dominance of a single dysregulated mechanism.

Collectively, these results illustrate how the TENSE framework can distinguish between biological contexts characterized by inflammation-dominated dysregulation (e.g., microglial activation and sepsis) and those dominated by genomic stress responses (e.g., radiation exposure). Such mechanistic differentiation is difficult to infer directly from lists of enriched pathways, highlighting the utility of summarizing pathway enrichment outputs into structured, interpretable dysregulation profiles. Table 4 summarizes DIRES and TENSE scoring across the use cases.

**Table 4:** Summary of use cases and classification by TENSE and DIRES.

Use cases	D_rlt	I_rlt	R_rlt	E_rlt	S_rlt	TENSE
AD CM1 neuronal	0	0.029	0	0	0.013	0.022
AD CM8 microglial	0	0.208	0	0	0	0.208
AD module brown	0	0.131	0	0	0	0.131
AD module turquoise	0.005	0.038	0	0	0.009	0.012
AD NFT vs. plaques	0	0.111	0	0	0.009	0.096
AD Pink	0	0	0	0	0	0
AD Yellow	0	0	0	0	0	0
IHM_A	0	0	0	0	0	0
IHM_B	0	0.217	0	0	0	0.217
RR_12 h	0.2	0	0	0	0	0.2
RR_4 h	0.2	0	0	0	0	0.2
RS_12 h	0.364	0	0	0	0	0.364
RS_4 h	0.044	0.044	0	0	0	0.044
SOM	0	0.4	0	0	0	0.4
TM10 RNA metabolism	0.044	0.013	0.033	0.013	0.002	0.025

Use cases are presented in alphabetical order. The “_rlt” suffix denotes the utilization of relative scores (i.e., $n_{i}$ , i ∈ D, I, R, E, S) for each DIRES category.

### 4.2 Limitations, caveats, and further development

In this study, the TENSE and, by extension, DIRES are defined as an alternative to arbitrary annotation of pathway enrichment data and forming decisions about the contribution and directionality of biological disorder in various settings. Rather than a comprehensive annotation of every possible process, it adopts a utilitarian approach, annotating hubs of disorder as aging progresses and providing a summary estimate that translates dysfunction as measurable relative disorder. The use cases explored indicate that TENSE and DIRES align with the biology and novel findings of the original studies, showing concordance in annotating data stemming from similar biological contexts and capturing the directionality of biological processes responding to exposures.

TENSE should be viewed within the appropriate context and limitations. First, it should be acknowledged that aging is a complicated process and even more so at the molecular level. The pathways selected to construct the TENSE workflow are hypothesis-driven rather than data-driven themselves. This issue is addressed by selecting hub processes that reflect molecular events that are not specifically modeled. For example, mtDNA leakage in the cytoplasm may not be directly modeled as a term in the formula; however, it serves as a proxy in the Nucleic Acid Sensing term. Similarly, genotoxic stress from various agents or metabolic/proteostatic effects is not directly modeled by a dedicated term but would correspondingly score as a proxy in the D, R, and I categories – as shown in the relevant use case 2.

Another caveat is that the theoretical formulation of TENSE involves inherent skew. For instance, the TENSE formulation processes D, I, R, E, S as additive and equivalent contributors. This potential caveat is addressed by substituting each constituent with its standardized equivalent and finally standardizing the output to its maximum value. This, in turn, yields interpretable values: higher TENSE values reflect greater relative utilization of the transcriptome, with DIRES revealing the relative contribution of each component. LLM stochasticity may produce TENSE values that are generally reliable based on our own iterative validation; however, the human consensus annotation herein is performed by the authors for proof of concept. A broader concept that utilizes multiple raters, e.g., a Delphi consensus and reporting inter-rater agreement statistics, can further bolster human-reference reliability and provide a more direct comparator to LLM stochasticity.

Similarly, it is worth reiterating, as a potential caveat, that TENSE does not capture the detailed contributions of each possible contributor to aging, but rather summarizes several salient hub processes. However, this limitation can be further overcome by adding additional hub functions in the DIRES schema, e.g., mitochondrial function, and adjusting the TENSE formulation correspondingly. As a modular workflow, TENSE relies on a semantic layer that can be adapted to a specific concept. Rather than simply representing the weighted standardized sum of activity scores and imposing the arithmetic mean as a constraint for each DIRES term, it aims to reduce skew by biologically similar overrepresented processes. We have opted to constrain potential scoring inflation of DIRES terms by infection terms that report on redundant gene networks, such as ribosomal proteins. Hence, these pathways do not score explicitly in DIRES unless referring to specific inflammatory processes during the course of infection.

Another potential caveat of the TENSE framework is that its initial validation was restricted to a small selection of reference conditions, specifically one interferon-associated module and one housekeeping module. Although this demonstrates that the method can work, it does not confirm its applicability to a wide range of biological scenarios. To address this issue, future studies should include a more diverse set of baseline and homeostatic conditions, enabling a thorough evaluation of the framework’s robustness and accuracy, especially in situations close to floor effects or lower TENSE states. The TENSE workflow should also be considered within the context and constraints of overrepresentation analyses, in that they represent enrichment or depletion of gene sets against our knowledge base, rather than *in vivo* evidence of pathway activity. As such, high-throughput datasets containing, e.g., methylomics, differential gene expression, and DNA damage marker data can provide alternative formulations of TENSE and benchmarking closer to the theoretical model, rather than the current enrichment pattern-based formulation.

Lastly, possible permutations that utilize the same theoretical model are possible. For instance, another pathway-centric approach could incorporate Shannon Entropy (Shannon, 1948), which estimates pathways related to aging and standardizes their impact over the background in sequencing experiments (Carels, 2024; Zenil et al., 2018). Furthermore, it should be noted that it was not conceptualized as a substitute or surrogate to existing predictors of biological age. Following Horvath’s clock (Horvath, 2013), several approaches utilizing data from epigenomics (Porter et al., 2021, Hannum et al., 2013, Prosz et al., 2024), metabolomics (Huang et al., 2025), transcriptomics (Zakar-Polyák et al., 2024) and proteomics (Argentieri et al., 2024) based approaches, among others (Galkin et al., 2020) have provided deeper insight into deriving accurate biological age estimates (Jansen et al., 2021). By design, TENSE and DIRES were constructed to capture a distinct biological signal from aging clocks. While a biological clock would be utilized to estimate biological age, TENSE would be utilized to estimate the effect of an exposure that maps to age-related processes, or the skew of any of these processes (e.g., inflammation and/or DNA damage) in a biological context (e.g., disease).

TENSE can thus complement these approaches, functioning more like a biological compass and accelerometer, rather than a biological clock.

## 5 Conclusion

The TENSE and DIRES scoring schema represented a novel, reductionist approach in annotating pathway enrichment data and an alternative to arbitrary or hypothesis-driven interpretation. The responsiveness of both TENSE and DIRES to complex disease processes, cell-specific gene modules, and complex exposures, including ionizing radiation, across diverse biological contexts, suggests further potential usage beyond aging-related disease. Using the provided cases, we highlighted the potential of TENSE as a reproducible and interpretable method for summarizing complex pathway enrichment outputs into biologically meaningful estimates of immune-aging–related dysregulation. In this sense, TENSE transforms pathway enrichment outputs from descriptive lists into quantitative, mechanistically interpretable estimates of system-level dysregulation.

## Data Availability

The original contributions presented in the study are included in the article/Supplementary material; further inquiries can be directed to the corresponding author/s.

## Footnotes

1 KNIME Workflow version 5.10; available from https://www.knime.com; Accessed March 9th 2026

2 Available from Raw data usage from referenced studies, Enrichr access to Reactome Library version 2024; Available from https://maayanlab.cloud/Enrichr/enrich; Accessed March 9th 2026

3
https://openai.com/index/gpt-4-1

4 Available from: https://developers.openai.com/api/docs/models/gpt-4.1-nano; Accessed 09/03/2026

5 Library version 2024; Available from https://maayanlab.cloud/Enrichr/enrich

6 version 5.10; available from https://www.knime.com; run locally
